# A study on the standard setting, validity, and reliability of a standardized patient performance rating scale – student version

**DOI:** 10.1080/07853890.2023.2168744

**Published:** 2023-01-30

**Authors:** Ipek Gonullu, Celal Deha Dogan, Sengul Erden, Derya Gokmen

**Affiliations:** a Department of Medical Education and Informatics, Ankara University Faculty of Medicine, Ankara, Turkey; b Department of Measurement and Evaluation, Ankara University Faculty of Education, Ankara, Turkey; c Department of Biostatistics, Ankara University Faculty of Medicine, Ankara, Turkey

**Keywords:** Performance rating scale, reliability, standard setting, standardized patient, student ratings, validity

## Abstract

**Introduction:**

The quality of the performances of standardized patients plays a significant role in the effectiveness of clinical skills education. Therefore, providing standardized patients with constant feedback is essential. It is especially important to get students’ perspectives immediately following their encounters with standardized patients. In the literature, there is no scale for use by students to evaluate the performance of standardized patients. Thus, the three main goals of this study were to: (1) develop a scale for use by students to evaluate the performance of standardized patients, (2) examine the psychometric properties of the scale, and (3) determine a cut-off score for the scale in a standard-setting

**Materials and methods:**

Seven hundred and two medical students participated in the scale- development process, the pilot test, and the validation process, and seven educators took part in the standard-setting process. After the evaluation of content validity, construct validity was assessed via exploratory and confirmatory factor analyses. For the standard-setting study, the extended Angoff method was used.

**Results:**

The exploratory factor analysis revealed that the scale had a single-factor structure, which was confirmed by confirmatory factor analysis. The Cronbach’s alpha internal consistency coefficient was 0.91. The scale consists of nine items. The cut-off score was determined to be 24.11/45, which represents the minimum acceptable standard for standardized patient performance.

**Conclusions:**

Our study outlined the critical steps in developing a measurement tool and produced a valid and reliable scale that allows medical students to assess the performance of standardized patients immediately following their interaction with the standardized patient. This scale constitutes an important contribution to the literature as it provides a tool for standardized patient trainers to assess standardized patients’ weaknesses and help them improve their performance.

**KEY MESSAGES**
- Evaluation of SP performance is essential to ensure the educational quality of clinical skills training programs.
- Students are the most relevant stakeholders to give feedback about SP performance immediately after encounters.
- The ‘Standardized Patient Performance Rating Scale – Student Version’ is a valid, reliable scale that can be used by students for the evaluation of standardized patients’ strengths and weaknesses at individual-performance levels quickly.

## Introduction

Standardized patients (SP) play an integral role in teaching communication and clinical skills in contemporary medical education. Howard Barrows was the first to use SPs in the 1960s [1–3]. SP programs, which are flexible and can be used with different teaching approaches, provide medical students with standardized learning opportunities and a learning environment in line with adult learning principles. SP encounters, which are exceptionally well suited for teaching and assessing student performance in a safe environment, allow students to overcome the fear of harming a patient before they are required to encounter actual patients [4–6].

It is important to monitor SP performance to ensure consistency and, in turn, the effectiveness of medical education. To maintain the quality of SP performance, a common quality assurance method is the analysis of recorded student–standardized patient interactions after the encounter [7]. In addition, faculty members are encouraged to complete brief written evaluations of SP performances, although they seldom provide feedback [8].

Faculty working with SPs [8], SP trainers, SPs [7,9], and students are the most relevant stakeholders in the assessment of SP performance. The results of a previous study showed that the evaluations of medical students and faculty of SP performance were similar, which suggests that the students were able to recognize the quality of the constructive feedback that they received from the SPs [10]. As medical students interact one-on-one with SPs, it is essential to get feedback from the students about the SP performance immediately following the encounters. Because more than one medical student assesses the performance of an SP at different times, it is possible to obtain comprehensive information and monitor the progress of SP performance over time. In addition, SPs can receive systematic feedback on their strengths and weaknesses, enabling them to deliver more effective and more consistent performances to enhance student-centered learning.

Like the performance of SP, psychological constructs are complex and difficult to measure. For this reason, having several tools that measure the same constructs contributes to the literature. In this way, users will be able to compare different measurement tools and choose the most suitable one. The literature contains several scales for evaluating SP performance. While some of these scales mainly focus on accurate portrayals of case specifics [11,12], others do not [9,13,14]. Some scales can be used by all stakeholders [9,10,13,14], but to the best of the authors’ knowledge, there is no existing scale for use specifically by medical students that does not focus on case specifics.

The existing scales that are used to assess SP performance include between 21 and 28 items [13,14], and thus, none of them is short enough to be used effectively following repeated encounters between medical students and standardized patients. In contexts where the number of SPs is high, and evaluations need to be completed quickly after each encounter, a scale with fewer items would be more practical.

It is crucial to establish cut-off scores when developing standard scales. Standard setting is the methodology for defining achievement and proficiency levels as well as for identifying cut-off scores corresponding to those levels [15]. If the cut-off scores are not appropriately set, the results of the assessment could be questionable. For this reason, standard setting is a critical component of the test development process [16]. A standard scale with a cut-off score facilitates decision-making about SP performance. No previous studies have developed a standard scale with a cut-off score specifically for medical students to assess the quality of standardized patients’ performances.

Consequently, developing a valid and reliable scale with fewer items specifically for medical students to evaluate SP performance in an educational setting and carrying out a standard-setting study for this scale will make a significant contribution to the literature. Therefore, this study has three main goals:

1. Develop a scale for use by medical students to evaluate the performance of SPs.
2. Examine the psychometric properties of this scale.
3. Conduct a standard-setting study to define a cut-off score for this scale.

## Methods

This methodological study used the three-step process to develop a scale to evaluate SP performance by students as follows:

Phase 1: development of the scale,
Phase 2: validation of the scale, and
Phase 3: standard-setting of the scale.

These three phases are explained separately in the sections Development of Data Collection Tools, Data Collection, and Data Analysis.

This study was approved by the Ethics Committee of Ankara University School of Medicine (AUSM) (approval number: 15 January 2016; date: 11 January 2016).

### Participants

Two groups of participants were involved in this study: Sample 1 was used in Phase 1 and Phase 2, and Sample 2 was used in Phase 3.

#### Sample 1

The medical curriculum at AUSM entails a 6-year program comprising 3 years of preclinical work followed by 3 years of clinical work (2 years of clerkship and one year’s internship). Communication training with SPs in preclinical years is a mandatory part of the curriculum, and SP encounters are conducted during the second and third years. For this reason, we included second- and third-year medical students in the 2016–2017 academic year. The criteria for participation in the study were having at least one previous SP encounter experience.

In total, 702 medical students participated in Phase 1 of the study. While determining the sample size, the requirements of multivariate data analysis [exploratory factor analysis (EFA) and confirmatory factor analysis (CFA)] were considered. As these are multivariate statistical methods, they require large sample sizes. According to Comrey and Lee, a sample size of 200 is fair, and a sample size of 300 is suitable for EFA [17]. Moreover, at least 300 cases are needed with low commonalities, a small number of factors, and just three or four indicators for each factor [18].

As EFA and CFA should be conducted with two different groups selected from the same population, we distributed the participants to each process. Second-year medical students performed the SP evaluation process earlier than third-year medical students did. Since EFA was performed earlier than CFA, EFA was done using the data of the second-year students (*n* = 307), and CFA was done using the data of the third-year students (*n* = 395).

#### Sample 2

The standard-setting study of the scale was carried out with a test-centered approach, following which expert opinions were collected. Experts with at least five years of experience in using and training SPs were selected through purposive sampling. Purposive sampling is a type of nonprobability sampling in which the researcher consciously selects specific elements or subjects for inclusion to ensure that the elements will have certain characteristics relevant to the study. In addition, while selecting the experts, it was considered that they work in different departments of the medical school, as they may have different perspectives. For this purpose, two SP trainers and faculty from the Department of Medical Education and five faculty members from the Infectious Diseases, Child Health and Diseases, Psychiatry, Radiology, and Forensic Medicine departments participated in this phase.

Consent was obtained from all the participants in both Sample 1 and Sample 2.

### Development of data collection tools

#### Phase 1

The scale development process comprised seven steps, namely a literature review, conducting interviews, synthesis of the literature review and interviews, developing items, consulting experts, preliminary application, and pilot testing [19].

##### Literature review

The keywords ‘standardized/simulated patient performance’ and ‘standardized/simulated patient scale’ were used on the Web of Science, Google Scholar, and ProQuest search engines to locate relevant literature. During this stage, the two research studies focusing on the development of measurement tools for SP performance evaluation were investigated [13,14] and eventually assessed. Both MaSP (Maastricht Assessment of Simulated Patients) and Nijmegen Evaluation of the Simulated Patient (NESP) were developed after structured interviews with medical students, experienced tutors, medical psychologists, physicians, and experts in the field of SPs [13,14]. They were asked what they considered the key features of good and poor SP performance in an educational setting. The analysis of the interviews for MaSP revealed that the two main variables determining the quality of SP performance were authenticity and feedback [13], whereas for NESP items they were to evaluate the performance of SPs on their ability to role play and provide feedback [14].

The domains of SP performance were defined in a well-known book written for coaching SPs as follows: the ability to portray a patient, to observe the medical student’s behavior, to recall the encounter, and to give feedback [20]. SPs must give accurate medical history and realistically depict the patient’s educational level, psychological state, as well as emotional condition while observing the student’s performance. After the interview, the SPs must recall the details of the student’s behavior and give thoughtful, beneficial, and effective feedback from the standpoint of the patient the SP was portraying. These conceptual definitions of the domains were decided to be measured for gauging the SPs’ performance.

##### Conducting interviews

In addition to the literature review, interviews were conducted with 9 faculty and 50 medical students (different from the participants in Phase 1 and Phase 2) and with two field experts who participated in SP training. Individual oral interviews of 15–30 min were made from among 45 faculty who had been involved in SP selection or working with SPs for at least 7 years, especially in communication skills training. They were asked what they considered the main attributes of good and poor SP performance. They focused on the different performance characteristics in this role, such as persuasion, successful portrayal, respecting the scenario, and giving effective feedback. When the answers started to repeat after nine tutors, interviewing was stopped. Both written and verbal responses were collected from these interviews.

##### Synthesis of the literature review and interviews

The data from the literature review and the interviews were together evaluated with the domains of SP performance. As a result, the scope and content of the measurement tool intended to be developed were determined, and four domains and nine conceptual definitions were identified.

##### Developing items

Over four domains (the ability to portray a patient, to observe the medical student’s behavior, to recall the encounter, and to give feedback), a pool of 18 items was created based on a synthesis of the literature review and the interviews. Two items were assigned to each conceptual definition in order to prevent narrowing of the scope of the scale in a situation where an item is removed as a result of expert opinion or item analysis (Table 1).

**Table 1.** Item pool.

Domain	Conceptual definition	Items
To ability to portray a patient	Persuasiveness of acting	The standardized patient plays the role realistically.
		The appearance of the standardized patient fits his/her role
	Portraying a patient	The standardized patient’s role is understandable.
		Before the feedback, the standardized patient gets out of the patient role.
	Acting according to the scenario	The standardized patient’s answers are appropriate to the questions
		The standardized patient does not provide unnecessary information during the interview.
To observe medical student’s behaviour	Observing the student performance	The standardized patient’s feedback is relevant to my performance.
		Throughout the interview, the standardized patient evaluates my performance appropriately.
To recall the encounter	Recalling the encounter	During the feedback session, the standardized patient gives specific examples from the interview
		The standardized patient gives feedback on behaviors other than medical issues.
To give feedback	Using communication skills while giving feedback	The standardized patient incentivizes me to ask questions during the feedback session.
		The standardized patient listens to me while giving feedback.
	Competency in giving feedback	During the feedback session, the standardized patient communicates how he/she felt as a patient during the interview.
		The interview increases confidence in terms of patient-physician communication.
	Efficacy of the feedback	During the feedback session, the standardized patient gives remedial feedback.
		The standardized patient’s feedback is useful.
	Professional attitude while giving feedback	The standardized patient gives feedback in a professional manner.
		The standardized patient uses appropriate language when giving feedback.

All developed items were positively worded. A 5-point Likert-type scale was determined in consultation with the experts (three medical educators and one measurement-evaluation specialist, excluding the experts who participated in the Consulting Expert Validation phase). The response anchors of these items were defined as ‘poor (1)’, ‘fair (2)’, ‘good (3)’, ‘very good (4)’ and ‘excellent (5)’. After these steps, a draft version of the scale was formed.

##### Consulting expert validation (content validity)

To obtain an opinion on the 18-item draft scale, seven experts working in the field (four volunteer faculty experienced in using and training SPs, two linguists, and one of the authors, who is a measurement-evaluation specialist) were consulted. These experts examined the scale items in the context of content, scope, language, comprehensibility, measurement, and evaluation principles by using an evaluation form. On the form, the experts stated their opinions on each item as ‘applicable’, ‘not applicable’, or ‘needs revision’ and subsequently included their recommendations for these items. Based on their recommendations, six items were excluded, and one item was revised; thus, a twelve-item pilot version of the scale was created (Table 2).

**Table 2.** The results of expert validation and pilot testing.

Domain	Conceptual definition	Items	Expert opinion			Pilot testing
To ability to portray a patient	Persuasiveness of acting	The standardized patient plays the role realistically.	Accepted as is			
		The appearance of the standardized patient fits his/her role	Accepted as is			Omitted because of low factor loading—0.19
	Portraying a patient	The standardized patient’s role is understandable.	Accepted as is			
		Before the feedback, the standardized patient gets out of the patient role.		Omitted		
	Acting according to the scenario	The standardized patient’s answers are appropriate to the questions	Accepted as is			
		The standardized patient does not provide unnecessary information during the interview.		Omitted		
To observe medical student’s behaviour	Observing the student performance	The standardized patient’s feedback is relevant to my performance.	Accepted as is			
		Throughout the interview, the standardized patient evaluates my performance appropriately.		Omitted		
To recall the encounter	Recalling the encounter	During the feedback session, the standardized patient gives specific examples from the interview	Accepted as is			
		The standardized patient gives feedback on behaviors other than medical issues.		Omitted		
To give feedback	Using communication skills while giving feedback	The standardized patient incentivizes me to ask questions during the feedback session.	Accepted as is			
		The standardized patient listens to me while giving feedback.	Accepted as is			Omitted because of low factor loading—0.23
	Competency in giving feedback	During the feedback session, the standardized patient communicates how he/she felt as a patient during the interview.	Accepted as is			
		The interview increases confidence in terms of patient-physician communication.		Omitted		
	Efficacy of the feedback	During the feedback session, the standardized patient gives remedial feedback.	Accepted as is			
		The standardized patient’s feedback is useful.		Omitted		
	Professional attitude while giving feedback	The standardized patient gives feedback in a professional manner.			Revised**:** ‘Standardized patient gives feedback in a kind manner’	
		The standardized patient uses appropriate language when giving feedback.	Accepted as is			Omitted because of low factor loading—0.18

##### Preliminary application

At this stage, the scale was applied to a group of 81 medical students (different from the participants in Phase 1 and Phase 2) in order to determine the approximate duration of implementation, to correct any incomprehensible items, and to make changes, where necessary. In this preliminary application, no item was misunderstood, none of the items were left unanswered, the instructions were comprehensible, and the evaluation of an SP took 3 to 5 min.

##### Pilot testing

The 12-item pilot form was applied to a large group of medical students (*N* = 702), following which the validity and reliability analyses comprising Phase 2 of the study were performed. After completion of these analyses, the scale was finalized. Relevant findings are presented in the Data Analysis section.

#### Phase 3

The extended Angoff method was used to determine the cut-off score for the scale. In this method, the experts estimated the number of scale points that they believed borderline examinees would obtain for each item [15]. In this context, experts determined the level of performance of the SPs at the borderline by using a ‘Standard-Setting Form’ for each item. In the form, two sections specified the level of performance of the SP at the borderline for each item. After carrying out discussions between the two sessions, the experts completed one of these sections in the first evaluation session and the other in the second evaluation session.

### Data collection

#### Phase 1

At AUSM, medical students practice interviews with SPs who are trained to act as patients with conflicts as well as a defined medical and life history. They have regular script training sessions for learning new roles or refreshing established roles and practicing the giving of feedback. Before entering the university SP pool, all SPs signed an ‘SP Commitment Form’ that included confirmation that materials related to them could be used for educational or research purposes. During SP encounters, SPs gave verbal feedback to the students from the patient’s perspective.

The Ethics Committee of AUSM approved the study. During the communication skills program, the medical students were informed about the study, and participation was voluntary. Before the interview with the SPs, two authors performed a 20-minute rater training for the volunteer medical students. The training consisted of information about the standards of SP performance, how to assess SPs immediately, and how to fill the scale. Consent was obtained before the interviews. In total, 25 SPs (6 male − 19 female, aged between 32 and 65 years**)** were utilized for the study.

The medical students were asked to respond to the scale immediately after the encounters. Each student evaluated the SP he/she interviewed only once during the communication skills program. Care was taken for the students to complete the scale alone without any interaction with their peers or others.

#### Phase 3

First, the experts were trained by the measurement and evaluation specialist. During this training, the aim and methods used in standard-setting were explained, and information was given about the procedures to be performed in the standard-setting method. In the next step, the experts discussed the characteristics they considered should be present in the SP and agreed on the level of competence that an SP at the borderline should have. After the first evaluation session began, the experts were asked to give scores (min: 1, max: 5) for each item by an SP at the borderline. This process was carried out individually. They were subsequently asked to share their evaluations with the group and justify them. Then, the experts stated their opinions about each other’s evaluations, which were followed by a discussion. In the second session, experts were asked once again to provide scores for an SP at the borderline. The duration of the first session, the discussion, and the second session was approximately 1 h. The reason for using two rounds in the Angoff method is to reduce deviation in the evaluations and to obtain results that are more reliable. The participants discussed their evaluations after the first round. The participants were given the opportunity to make changes in their evaluations in the second round because of these discussions if they deemed it necessary.

### Data analysis

#### Phase 2

##### Validity of the scale

###### Construct validity

Before EFA, Kaiser-Meyer-Olkin (KMO) and the Bartlett test of sphericity were examined, and the data were tested for appropriateness for the factor analysis. Bartlett’s test was applied to determine if the correlation matrix was different from the identity matrix. The statistical significance of the calculated chi-square value in the Bartlett test can be interpreted as the data are appropriate for factor analysis [21].

The principal components method was used in the factor selection process. It has been reported that Kaiser’s criterion is too strict and, consequently, the criterion often overestimates the number of factors [22,23]. In the determination of the number of factors, scree plot and parallel analysis methods were used. Since the scale had a single factor structure, no rotation method was used. Horn’s parallel analysis is one of the most common methods used to decide the number of factors. The parallel analysis compares each eigenvalue against an eigenvalue for the corresponding factor in many randomly generated data sets. These generated data sets have the same characteristics as the data being analyzed. In doing so, each eigenvalue is being compared to an eigenvalue from a data set that has no underlying factors [24].

In the CFA process, as the data did not satisfy the multivariate normality assumption, the analysis was carried out based on the weighted least squares method, and the standardized coefficients, corresponding *t* values, and some fit indices were evaluated. While a ratio of chi-square value to the degree of freedom of below 2.5 indicates perfect fit, the corresponding values for the non-normed fit index (NNFI), comparative fit index (CFI), goodness of fit index (GFI), and adjusted goodness of fit index (AGFI) are above 0.95. For the root mean square error of approximation (RMSEA) and standardized root mean square residual (SRMR), values below 0.05 show perfect fit [25].

###### Item discrimination

In order to assess item discrimination, the significance of the differences between the scores of the participants in the upper and lower 27% groups for each item was compared using the Mann–Whitney *U* test. Since the scores given for each item were within the ranking level, parametric tests (*t*-test for independent groups, etc.) were not used in this comparison.

##### Reliability of the scale

To assess the reliability of the developed scale, Cronbach’s alpha [26], the internal consistency coefficient, and the split-half reliability coefficient [27] were calculated. The test-retest reliability coefficient could not be calculated because it was practically impossible to reach the participants twice. Since a large number of medical students evaluated SPs in this study, inter-rater reliability was not calculated because it would not be practical [28].

#### Phase 3: Standard setting of the scale

The methods commonly used in standard settings can be classified as test-centered and exam-centered. Angoff, which is a test-centered standard-setting approach, is a widely used and practical method. With this method, a cut-off score can be determined before the test is administered. In examinee-centered methods (e.g. borderline regression method, constricting groups method), the cut-off score is determined after the test is applied. In order to use this method, the experts involved in the standard-setting process need to be familiar with all the SPs because they have to classify their performances as successful or unsuccessful. Since the experts in this study did not know the SPs well enough, a decision was taken to use the Angoff method, a test-centered approach. In addition, test-centered and examinee-centered standard-setting methods give similar results, if applied correctly.

An adaptation of the Angoff method for items with more than two possible scores is called the extended Angoff method [29]. Candidates at the borderline are those at the sufficient-insufficient border and those who are considered barely sufficient. Using the extended Angoff method, the experts decided the scores for each item of the SP at the borderline and recorded these estimates. The mean of the estimates given by the experts was calculated for each item. The sum of means gave the cut-off score.

During the data analysis process, the SPSS 21.0, Lisrel 8.7, Excel 2016, and Monte Carlo PCA for Parallel Analysis packages were used.

## Results

### Phase 1: Development of the scale

Development of the scale was described in detail based on the seven-step process presented by the Association of Medical Education in Europe Guide in the Development of Data Collection Tools section. Upon completion of the preliminary application, which is the sixth step of the above-mentioned process, a 12-item pilot form was created.

### Phase 2: Validation of the scale

#### Validity of the scale

##### Construct validity

###### Exploratory factor analysis

EFA was carried out on 307 participants. The KMO value for this study was calculated as 0.92. The results of the Bartlett test indicate that a chi-square value of less than 0.05 is significant, which, in turn, shows that the data are appropriate for factor analysis [30].

As a result of EFA, the scale had three factors with an eigenvalue greater than 1. When more than 200 samples are reached, it is recommended to examine the scree plot in accordance with the eigenvalues for determining the number of important factors [31]. There is a significant deceleration in the scree plot after the first factor, and the rate of deceleration decreases and follows a horizontal course after the second factor. Moreover, the eigenvalue of the first factor (5.432) is approximately five times greater than the eigenvalue of the second factor (1.082) before the rotation. It has been suggested that a ratio of first-to-second eigenvalues greater than four is evidence of unidimensionality [32,33]. The first factor alone, yielding a high variance (45%), was interpreted as the scale having a single-factor structure. The parallel analysis for the factor number also supports the single-factor structure.

After establishing that the scale had a single-factor structure, the analysis was performed once again. The single-factor structure explained 59% of the total variance. Since the scale had a single-factor structure, no rotation was performed. In the analysis process, three items with a factor loading value below 0.40 (item 4, 0.19; item 5, 0.18, and item 7, 0.23), were excluded starting from the item with the smallest factor loading (Table 2). The nine items and the factor loading values for the rest of the scale are presented in Table 3.

**Table 3.** SPRS-S items and factor loading values.

Items	Factor loading value
The standardized patient plays the role realistically (1).	0.729
The standardized patient’s role is understandable (2).	0.744
The standardized patient’s answers are appropriate to the questions (3).	0.789
The standardized patient’s feedback is relevant to my performance (4).	0.824
During the feedback session, the standardized patient gives specific examples from the interview (5).	0.723
The standardized patient incentivizes me to ask questions during the feedback session (6).	0.720
During the feedback session, the standardized patient communicates how he/she felt as a patient during the interview (7).	0.773
During the feedback session, the standardized patient gives remedial feedback (8).	0.812
The standardized patient gives feedback in a kind manner (9).	0.815

Communality values were examined in the EFA process. It was determined that all the communality values of the items were above 0.50. According to the result, the scale had a single factor structure and consisted of nine items. It was named the ‘Standardized Patient Performance Rating Scale – Student Version (SPRS-S)’.

###### Confirmatory factor analysis

CFA was performed for verification of the single-factor structure resulting from the EFA of the SPRS-S developed within the scope of the study. As a result of CFA on the single-factor structure of the SPRS-S, the t values of the latent variables related to observed variables were greater than the critical value (2.58) and statistically significant at the 0.01 level. In the analysis, the software suggested that the errors related to the items s1 and s2 should be associated and that this association could result in a decrease of 26.91 in the chi-square value. This theoretically reasonable modification was accepted, and errors of items s1 and s2 were associated. Figures containing standardized coefficients and *t* values for the model are given as Supplementary Material.

After that, the ratio of chi-square value to the degree of freedom was calculated as 1.81. This value can be considered an indicator of perfect fit [18]. Other calculated indices are as follows: NNFI, 0.97; CFI, 0.98; GFI, 0.97; AGFI, 0.96; RMSEA, 0.04; and SRMR, 0.04. When the results are examined, the values of all fit indices are indicative of perfect fit [25].

###### Item discrimination

For each item included in the scale, the mean ranks were higher in favor of the group in the upper 27%, and these differences were significant (*p* < 0.01).

#### Reliability of the scale

Cronbach’s alpha internal consistency coefficient was calculated as 0.91 and the split-half reliability coefficient as 0.87. These findings show that the internal consistency coefficient of the scale is at the desired level. A Cronbach alpha coefficient of 0.80 or above indicates that the test has a high level of internal consistency [34].

### Phase 3: Standard setting of SPRS-S

Seven experts were consulted in order to determine the cut-off score of SPRS-S (nine items). The experts were asked to use the extended Angoff method and give a score between 1 and 5, taking into account an SP at the borderline for each item. This scoring was undertaken in two rounds (R), as shown in Table 4.

**Table 4.** Expert ratings obtained from the extended Angoff method.

Expert ID		Item number									
		1	2	3	4	5	6	7	8	9	Row means(SD)
1	Round 1	3	4	4	3	3	2	3	4	4	3.33 (0.70)
	Round 2	3	3	2	2	3	3	3	4	4	3.00 (0.70)
2	Round 1	4	3	4	3	4	3	4	3	4	3.56 (0.52)
	Round 2	4	4	3	3	4	4	4	4	4	3.78 (0.44)
3	Round 1	4	4	4	2	3	3	4	3	4	3.44 (0.72)
	Round 2	4	4	4	3	3	3	4	3	4	3.56 (0.52)
4	Round 1	3	4	4	3	4	3	3	3	4	3.44 (0.52)
	Round 2	4	4	3	3	3	3	3	3	4	3.33 (0.50)
5	Round 1	4	4	4	4	4	5	4	4	5	4.22 (0.44)
	Round 2	4	4	3	4	4	4	4	4	5	4.00 (0.50)
6	Round 1	3	3	4	2	2	2	3	2	3	2.67 (0.70)
	Round 2	3	3	4	2	3	3	3	3	4	3.11 (0.60)
7	Round 1	3	3	3	4	4	3	4	3	4	3.44 (0.52)
	Round 2	3	3	3	3	4	3	4	3	4	3.33 (0.50)
Round 1 means		3.43	3.57	3.86	3.00	3.43	3.00	3.57	3.14	4.00	3.44 (0.59)
Round 1 SD		0.53	0.53	0.38	0.82	0.79	1.00	0.53	0.69	0.58	
Round 2 means		3.57	3.57	3.14	2.86	3.43	3.29	3.57	3.43	4.14	3.44 (0.53)
Round 2 SD		0.53	0.53	0.69	0.69	0.53	0.49	0.53	0.53	0.38	

SD: standard deviations; Round 1 means: column means for round 1; Round 1 SD: column standard deviations for round 1; Round 2 means: column means for round 2; Round 2 SD: column standard deviations for round 2.

When the cut-off scores for each item were examined after the second round, the experts allocated the lowest cut-off point to item 4 (2.857) and the highest cut-off point to item 9 (4.14). Also, the experts stated that an SP at the borderline should have an average of 3.44 points from each item. The standard deviation values were examined to determine the variability between the experts’ scores, and the variability between expert opinions was smaller in round 2 (0.53) than in round 1 (0.59).

The mean of the total points of each expert for each item was taken to calculate the cut-off score. Then, these means were summed to obtain the cut-off score.

Cut-off score=3.00+3.78+3.56+3.33+4.00+3.11+3.33

Cut-off score=24.11

Therefore, according to the findings from the extended Angoff method, for an SP to be sufficient, he/she must obtain at least 24.11 out of 45 on SPRS-S.

## Discussion

As student evaluation of teaching ratings serve as a source of feedback for instructional improvement [35], the overall purpose of our study was to develop a valid, reliable instrument used only by students to evaluate SP performance. By using this scale, standardized patients can assess and address their weaknesses at an individual-performance level more easily and in a timely manner. It also can give hints to standardized patient trainers about the situations to be considered.

SPs might be more frequently used in summative or high-stakes assessments where they function more as an examination question than for educational purposes. But in some programs, they are used for educational purposes more than for summative assessments. For example, in Maastricht their rate of use of SP in high-stakes assessment vs. use for educational purposes is 15–85% [13]. In learning settings, standardization is less critical, and often its absence can be a feature. The tailoring of SP encounters can be used to meet the needs of individual learners [36]. Programs where SP encounters take place for educational purposes, particularly where feedback is a major formative assessment, student evaluations are a useful tool for student satisfaction and improvement of instruction, teacher, or SP.

The authenticity of role-playing and the quality of feedback provided by SPs are of high importance for quality of learning during SP contact learning sessions [13,14]. In this sense, assessing the individual performances of SPs in an educational setting and assisting them in addressing their weaknesses can improve the quality of a clinical skills training program. In addition, summative assessment, like Objective Structured Clinical Examination (OSCE), uses SPs who could cause measurement errors [37]. As a result, it is important to consider the training of SPs and monitor their performance and development. The performance of SPs may only be assessed from recorded videos, mostly by SP trainers and sometimes by faculty or students. On the other hand, assessing the performances of individual SPs from video systematically and giving feedback on these performances requires time. Furthermore, it is very difficult to assess all the records. We might be able to solve this problem by involving students in the assessment of an SP performance immediately after the encounter. Real-time feedback for SPs to improve their work can be easier and more useful than evaluating their performance from recorded videos.

In this study, we presented a unique evaluation tool filled out by medical students immediately after their encounter with SPs. The SPRS-S was developed, and validity, reliability, and standardization analyses were performed. As a result of EFA, it was determined that the scale had a single-factor structure confirmed by CFA. The nine items of the SPRS-S cover both four predefined domains and their conceptual definitions, which indicates content validity. Due to the aforementioned structure, the SPRS-S measures SP performance. It is recommended that future users adopt the scale as one-dimensional without considering the subdomains. The internal consistency of the scale was shown to be at the desired level in reliability analysis. The scale consists of nine items scored out of 5, implying that the lowest achievable score is 9 and the highest is 45. The cut-off score of an SP was 24.11 (out of a total of 45) determined by the extended Angoff method. In this context, for someone to successfully qualify as an SP, he/she must obtain at least 24 points on the SPRS-S. If he/she fails, an extra SP education program should be applied.

Literature supports that students can adequately assess the value of the education they receive [38], and they are critical stakeholders in medical education, for which their engagement is a vital component [39,40]. They offer a unique perspective that adds value to curricular issues and intangibles of the learning environment, which may be opaque to educators [39,40]. Involving students as key stakeholders in their education can have a profound impact on students and the institutions that serve them [39]. During the assessment of the SP performance, straightforward and intuitive perspectives could be valuable (even essential) to consider, as students interact with SPs one-on-one. The assessment of SP performance by students might have certain advantages compared to assessment done by other stakeholders. For example, several students can assess SPs, whereas only a few faculty members and even fewer SP trainers can. Furthermore, students can assess SPs at different times, so it is possible to monitor SP performance progress over time. Moreover, this scale can be used to identify SPs who need further training early on by picking out ones who score less than 24, which will bring efficiency to the SP training and development process.

In order to determine whether the performance of an SP is qualified, there should be more than one student evaluation. Although it is not known exactly how many interviews will be evaluated, as many assessments as possible should be done before a judgment is made related to SP qualifications. This number could be clarified in future studies.

The strength of this study is that the SPRS-S is not a case-specific scale and can be used for various scenarios. Due to the low cut-off score, the weak and strong performance of SPs can be defined easily and immediately. We recommend that if it is possible, the SPRS-S must be used by students immediately following an encounter for time-saving purposes because it is short. However, if it is not convenient to assess immediately, it can be conducted at a later time point as well. It can easily be completed by students in 3–5 min. The SPRS-S is personalized for students; it contains items related to their interactions with SPs, and it has a cut-off score, which is a critical component of the test development process.

One of the limitations of the study is that criterion validity cannot be tested because there is no other reliable, valid, and short scale for students to assess SP performance. However, in similar studies, the SPRS-S can be used as a criterion scale to test validity. The other limitation is instead of having two separate groups consisting of a mix of second- and third-year medical students, we used second-year students in the EFA calculation process and third-year students in the CFA calculation process. This decision assumed that second- and third-year medical students have very similar characteristics. In addition, second-year medical students performed the SP performance evaluation process earlier than third-year medical students did.

Since all experts expressed very similar opinions during the content validity examination of the scale, the content validity index and ratio were not calculated. However, the inability to calculate this index can be considered a limitation of the study.

Another limitation may be the presence of recall bias among students during their evaluation of SP performance. For this reason, the scale may not be used after summative assessments. Over half the questions on the scale represent the ability to give feedback. This limits the utility of the SPRS-S to only formative role-playing sessions, and it cannot be used in scenarios such as OSCEs in which students are not given verbal feedback at the end of the session. As there is no comparison between students and other assessors for the evaluation of SP performance, the results depend on only student perceptions, and this can also be considered a limitation of the study. We recommend that future investigations compare student ratings with those of other assessors to provide evidence for validity/reliability. But SPs who get under the cut-off point can be immediately evaluated by SP trainers and, if needed, they can be assigned an SP education program. Further, in addition to the nine items of the SPRS-S, there was no space for narrative assessment. This could be more helpful for the SP to learn areas in need of improvement.

There is an important point to be considered in the use of SPRS-S. When an SP gets full points from half the items, it reaches the cut-off point even if he/she gets zero points from the other half. In this case, the SP may seem sufficient even if it is very lacking in some dimensions. This is a limitation of both the Angoff method and classical test theory. In order to overcome this problem, computer-based and item response theory-based scaling are required. Practitioners who use the SPRS-S are advised to check whether SPs have received a certain score for each item, as well as the cut-off score obtained by the SP. In this context, researchers are recommended to carry out studies to determine cut-off points by using different methods for the SPRS-S.

## Conclusion

To ensure the educational quality of a program wherein SP encounters take place, evaluation of SP performance is important. This study presents a unique addition to SP training by introducing a student evaluation tool for SP performance immediately after encounters. Use of the SPRS-S, which has been confirmed for validity, reliability, and standard-setting in our analyses, will guide SP trainers during their SP training and their continued education post-training. It will also help SPs assess and address their weaknesses at an individual-performance level. For further studies using the SPRS-S, we recommend that researchers re-assess validity and reliability by using CFA and the internal consistency coefficient. In addition, the scale can be modified for other stakeholders, who can use it to assess SP performance. In conclusion, students can use this scale for the evaluation of SPs in the field of health sciences.

## Supplementary Material

Supplemental Material

## Data Availability

The data that support the findings of this study are available from the corresponding author, [DG], upon reasonable request.
