# Supplementary figures and images for: A study on the standard setting, validity, and reliability of a standardized patient performance rating scale – student version
Source: Ann Med. 2023 Jan 30;55(1):490–501. doi: 10.1080/07853890.2023.2168744 (PMC9888448; doi:10.1080/07853890.2023.2168744)

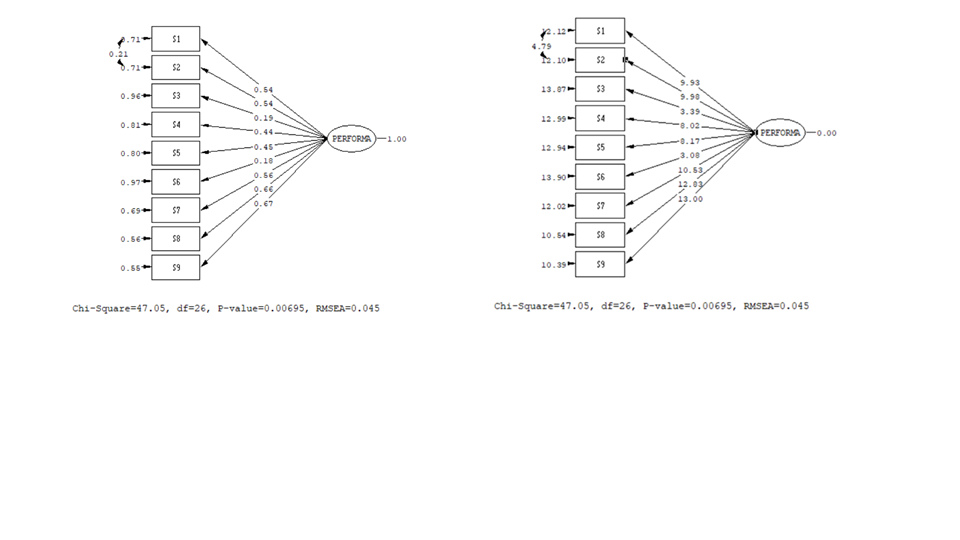

Supplement: Supplemental Material [file IANN_A_2168744_SM4815.png]
